# Role of pyrite in formation of hydroxyl radicals in coal: possible implications for human health

**DOI:** 10.1186/1743-8977-3-16

**Published:** 2006-12-19

**Authors:** Corey A Cohn, Richard Laffers, Sanford R Simon, Thomas O'Riordan, Martin AA Schoonen

**Affiliations:** 1 Department of Geosciences and Center for Environmental Molecular Science, Stony Brook University, Stony Brook, NY 11794-2100, USA; 2 Department of Pathology, Stony Brook University Hospital, Stony Brook, NY 11794, USA; 3 Department of Medicine, Stony Brook University Hospital, Stony Brook, NY 11794, USA; 4 National Institute of Occupational Health, Lerso Parkalle 105, 2100 Copenhagen, Denmark

## Abstract

**Background:**

The harmful effects from inhalation of coal dust are well-documented. The prevalence of lung disease varies by mining region and may, in part, be related to regional differences in the bioavailable iron content of the coal. Pyrite (FeS_2_), a common inorganic component in coal, has been shown to spontaneously form reactive oxygen species (ROS) (i.e., hydrogen peroxide and hydroxyl radicals) and degrade nucleic acids. This raises the question regarding the potential for similar reactivity from coal that contains pyrite. Experiments were performed to specifically evaluate the role of pyrite in coal dust reactivity. Coal samples containing various amounts of FeS_2 _were compared for differences in their generation of ROS and degradation of RNA.

**Results:**

Coals that contain iron also show the presence of FeS_2_, generate ROS and degrade RNA. Coal samples that do not contain pyrite do not produce ROS nor degrade RNA. The concentration of generated ROS and degradation rate of RNA both increase with greater FeS_2 _content in the coals.

**Conclusion:**

The prevalence of coal workers' pneumoconiosis can be correlated to the amount of FeS_2 _in the coals. Considering the harmful effects of generation of ROS by inhaled particles, the results presented here show a possible mechanism whereby coal samples may contribute to CWP. This suggests that the toxicity of coal may be explained, in part, by the presence of FeS_2_.

## Background

The occupational risks associated with coal mining are well-documented and range from entrapment in collapsed mining structures to health problems associated with chronic exposure to coal dust (see [1] for review of the hazards). The most common diseases encountered among coal miners are coal workers' pneumoconiosis (CWP) [2] and chronic obstructive pulmonary disease (COPD) [3]. While it is clear that these lung diseases stem from inhalation of coal dust, (with concurrent tobacco also contributing to COPD) it is not clear which component or components in coal cause, or contribute to disease. Coal is a variable mixture of organic carbon and inorganic minerals, such as quartz, clays, carbonates and pyrite [4]. The causes of coal-induced pulmonary disease are complex but several endpoints are used in studies to evaluate the mechanisms of toxicity and compare different coals for their toxicological potential. These include cytotoxicity, genotoxicity, generation of reactive oxygen species (ROS) and signs of oxidative stress, chemokine, cytokine, and growth factor release [5-7]. Given the well-documented correlation between exposure to quartz dust and lung disease [8-10], some studies have sought to correlate the prevalence of pulmonary inflammation, antioxidant production and radiographic small opacities [9] in coal miners to the quartz content in coals. While some studies support this notion that CWP is primarily due to exposure to quartz, there are other studies that show that a low prevalence of CWP occurred even among miners who had worked in a coal mine with high levels of quartz (for review see [8]). In other studies, the presence of minerals other than quartz has been noted to be critical for particle inflammatory potential [11]. For example, high iron concentrations in biological systems (e.g., iron released by ferritin under pathological conditions) can cause oxidation to biomolecules [12-16] and iron associated with asbestos [17,18], quartz [19], iron oxides [20,21], and iron sulfides [22-24] has been shown to be a key reactant in the mechanisms leading to lung injury.

**Figure 1:**
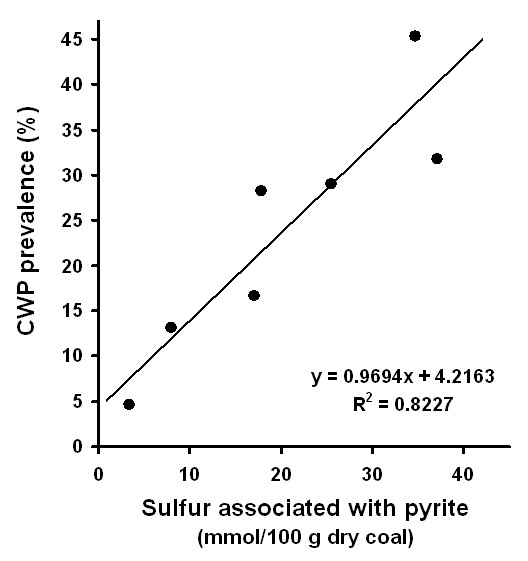
Correlation between sulfur that is associated with pyrite in coal and prevalence of coal workers pneumoconiosis. The data is from Huang et al., 2005 [25] who have compiled extensive data from the USGS coal quality database [53] and the first National Study of Coal Workers' Pneumoconiosis [54].

Epidemiological studies in the United States show a correlation (r = 0.94, p < 0.0015) between bioavailable iron (iron that dissolves in 10 mM phosphate solution at pH 4.5 under conditions which mimic the interior of lysosomes) released from coal and prevalence of CWP [25]. For example, coal miners from regions in the northeastern states, such as Pennsylvania where bioavailable iron values are high, developed more CWP, COPD, and asthma, compared to miners in western states such as Utah where bioavailable iron levels are low [25]. Experimental results show that coal samples with higher concentrations of bioavailable iron from Pennsylvania [26] induce higher levels of a pro-inflammatory cytokine [27] and greater alterations of lung cell genes [28] compared to coals from Utah. The epidemiological evidence shows little correlation (r = 0.28, p < 0.54) between quartz content and CWP [25]. This suggests that the presence of iron-containing minerals in coal may be a potentially more relevant factor in the development or progression of lung pathology than the presence of quartz. Iron in coal is associated predominantly with sulfur as FeS_2 _phases [29,30]. Many sulfur-rich coals contain iron disulfide (FeS_2_) in the form of pyrite or its dimorph, marcasite (hereafter we will use pyrite to indicate all forms of iron disulfide) [31]. When the pyritic sulfur in coal samples is plotted with CWP prevalence the resulting graph shows a correlation (Fig. 1). Although the presence of pyrite may correlate to CWP prevalence, the mechanisms whereby pyrite may lead to CWP have not been evaluated.

Pyrite has recently been shown to spontaneously generate hydrogen peroxide (H_2_O_2_) [32,33] and hydroxyl radicals (^•^OH) [23,24] when placed in water. The formation of these reactive oxygen species (ROS) also explains the recent observation that aqueous pyrite slurries degrade yeast RNA, ribosomal RNA, and DNA [24]. Pyrite is thought to form H_2_O_2 _through the iron-catalyzed Haber-Weiss reactions (eqs 1 and 2). The H_2_O_2 _can then react with ferrous iron dissolved from pyrite or at the pyrite surface to form ^•^OH [24] in the Fenton reaction (eq. 3). The iron in each of these reactions may be dissolved or surface-bound as these reactions can occur in solution or on the pyrite surface.

Fe(II) + O_2 _→ Fe(III) + (O_2_^•^)^- ^    (1)

Fe(II) + (O_2_^•^)^- ^+ 2H^+ ^→ Fe(III) + H_2_O_2 _    (2)

Fe(II) + H_2_O_2 _→ ^•^OH + OH^- ^+ Fe(III)     (3)

The overall process can be catalytic as the ferric iron formed in the reactions can be reduced by superoxide or another reducing agent, e.g., ascorbic acid [34], (i.e., reverse of eq. 1). In pyrite slurries, ferric iron can also be reduced by a reaction with pyrite. The reaction between ferric iron and pyrite is rapid and thought to be an important reaction in the weathering of pyrite [35,36].

While H_2_O_2 _is not very reactive toward nucleic acids [37], ^•^OH is considered to be a highly reactive species which attacks a wide range of molecules in aqueous solution at diffusion limited rates [38]. Hydroxyl radicals react with nucleic acids by hydrogen abstraction at the sugar or addition to the bases, both resulting in new radical moieties and de-polymerization [39,40]. Inhaled particle-induced formation of ^•^OH has been associated with genotoxicity [41,42] and oxidative stress [42,43]. Hence, ^•^OH formation *in vitro *and *in vivo *has been used as an indicator for mineral-induced toxicity potential [37,41-45].

Experiments have never been performed to determine if the pyrite fraction of coal will generate ^•^OH spontaneously. Earlier work has shown that ^•^OH is formed as a result of Fenton chemistry (eq. 1) when exogenous H_2_O_2 _is added to a coal sample that contains iron [46]. However, based on our earlier work we hypothesize that the pyrite fraction in coal will generate ^•^OH spontaneously (i.e., without addition of H_2_O_2_) and that coal samples containing higher levels of iron, i.e., more pyrite, will generate more ^•^OH. We speculate that pyrite-induced ROS may play an important role in creating and sustaining an imbalance in ROS within lungs of coal miners, which leads to chronic inflammation and increases the risk for diseases associated with inhalation of coal dust. In addition to ROS formed by particle/aqueous reactions, cells may also form ROS (e.g, H_2_O_2_) as a result of exposure to particles [47].

In order to evaluate the role of pyrite in coal generation of ROS and RNA degradation, we used a multi-inquiry approach. Coal samples containing various amounts of pyrite were examined for the presence of pyrite and compared for differences in their capacity to generate H_2_O_2_, ^•^OH, and degrade RNA in aqueous solutions. Table 1 lists the coal samples, their origins, type, total sulfur (i.e., sulfur oxy-anions, metal sulfides, and pyrite), BET surface areas, mercury mass fraction, and the concentration of iron released into solution after 7 hour exposures.

**Table 1:** Coal Samples

Sample #^a^	Origin	Coal type	Surface area (m^2^/g)^b^	Sulfur (mass fraction)^c^	Pyritic sulfur (mass fraction)^c^	Pyritic sulfur content per surface area (g/m^2^)	Iron release (μM)^d^
1635	Erie, Colorado	subbituminous	1.79	0.3616	0.00	0	0.00
2682b	Gillette, Wyoming	subbituminous	4.94	0.4917	0.01	0.002	0.02
2692b	Holden, West Virginia	bituminous	1.35	1.17	0.49	0.363	0.48
2684b	Marion, Illinois	bituminous	2.11	3.076	0.52	0.246	8.31
2685b	Captina, West Virginia	bituminous	1.72	4.73	1.15	0.669	13.3

^a ^National Institute of Standards & Technology sample numbers

^b ^BET surface area values are for coal as received and as used in experiments, not dessicated.

^c ^dry basis

^d ^0.125 m^2^/L coal loadings, total iron [i.e., Fe(II) & Fe(III)] after 7 hrs in 1.5 mg/L RNA solution

## Methods and materials

### Coal and pyrite samples

Coal samples were purchased from the National Institute of Standards & Technology (NIST). While this represents only a limited set of coals, the advantage is that these coals are thoroughly characterized and available to other researchers. BET surface area measurements with N_2 _were performed on the samples as received, which contained water ranging from 1 to 12% by mass. Experiments were performed with samples as received. All batch experiments (described below) were conducted with equivalent surface areas, so that all results could be directly compared. Pyrite crystals (Huanzala, Peru) were purchased from Ward's Natural Science (Rochester, NY) and crushed in an agate mill and sieved. The 63 to 38 micron fraction was stored in a vacuum desiccator until used in experiments (roughly six months after crushing).

### Scanning electron microscopy and energy-dispersive X-ray analysis

A LEO (now Nano Technology Systems Division of Zeiss) 1550 scanning electron microscope (SEM) equipped with a Schottky Field Emitting Gun was used in conjunction with a Phoenix/Sapphire energy-dispersive X-ray (EDAX) system for generating images of the surface and chemical composition, respectively. Samples were pressed onto carbon tape on aluminum mounts and examined. The experimental parameters are listed on the figure images.

### H_2_O_2 _quantification

H_2_O_2 _was quantified using leuco crystal violet as described elsewhere [33]. Leuco crystal violet (Spectrum) in the presence of the enzyme horseradish peroxidase (HRP) (Aldrich type II) forms a crystal violet cation, which has an absorbance maximum at 590 nm. Calibration curves were used to quantify the effects of H_2_O_2_, pH, aqueous ferrous iron salts (ferrous ammonium sulfate, Fisher), and EDTA (Fisher) on the absorbance of CV^+^. Catalase (from bovine liver, specific activity 40,000 to 60,000 units/mg, Sigma) was used at a concentration around 100,000 units per sample vial to verify the presence of H_2_O_2_. In a typical experiment, coal was weighed into a 4-mL methylcrylate cuvette and mixed with water containing 1 mM EDTA at pH 9 for two minutes followed by filtration (Millipore 0.45 μm) into another cuvette. Reagents [all stored at 4°C and brought up to room temperature (22 ± 2°C) before analyses] were then added to the aqueous filtrate in the following order, and at the indicated final concentrations, in a total volume of 2 ml: 100 mM KH_2_PO_4 _pH 4 buffer, 41 μM leuco crystal violet (from a stock solution of 31 mg LCV, 30 ml H_2_O, and 19.2 ml of 0.25 N HCl), and 0.5 units/mL HRP (50 μL from a stock solution of 4.5 mg HRP in 50 ml H_2_O). Samples were kept in the dark at room temperature (22 ± 2°C) for 30 minutes, upon which absorbance stabilized. Absorbance measurements were conducted in 1 cm path-length cuvettes and corrected for absorbance in a blank. The zero-H_2_O_2 _blank consisted of DI with all reagents in the proper concentrations.

### ^•^OH quantification

For the detection of hydroxyl radicals (^•^OH), 3'-(*p*-aminophenyl) fluorescein (APF, Invitrogen) was used. APF is not fluorescent until the aminophenyl group is eliminated from fluorescein by oxidation with ^•^OH, peroxynitrite anions (ONOO^-^) [48], or by horseradish peroxidase (HRP)-catalyzed oxidation with H_2_O_2_. The reactivity of APF is selective for only the very highly reactive oxygen species such as hydroxyl radicals and peroxynitrite anions and once reacted, forms a stable fluorescent product [48]. Results from experiments when catalase and radical scavengers (i.e., ethanol) are added to Fenton reagents (eq. 3) show a reduction in the fluorescence of APF (published separately). A calibration curve was first generated by reacting a series of nM concentrations of H_2_O_2 _with 10 μM APF in the presence of 2.95 units/mL (equivalent to 0.2 μM) HRP in 10 mM potassium phosphate buffer at pH 7.40 in a 4-mL methylcrylate cuvette (Fisher). These solutions were incubated in the dark at room temperature (22 ± 2°C) for 30 minutes and followed by fluorescence measurements (excitation/emission 490/520 nm, Barnstead Turner). In experiments performed to evaluate the generation of ^•^OH from coal slurries, coal samples were mixed with 10 μM APF in 10 mM potassium phosphate buffer, pH 7.40 in 2-mL vials and rotated end-over-end for 24 hrs in the dark. The suspensions were filtered through 0.45 μm filters and the fluorescence was measured (excitation 490 nm, emission 520 nm). The fluorescence data are presented on the figures as ^•^OH_APF_. Three replicate determinations with the same coal and pyrite composition were performed to estimate the uncertainty of the APF method.

### RNA stability assay

The capacity of the coal samples to degrade yeast RNA was evaluated by determining the decline in the concentration of full-length short strand RNA by loss of dye-binding capacity. This method has been described in detail elsewhere [49]. Yeast RNA (Spectrum) was prepared by making a saturated solution. This saturated solution was subsequently filtered through a 0.45 μm PVDF Millipore filter and the total RNA concentration was determined on the basis of its absorbance at 260 nm. The approximate length of the yeast RNA used in these batch experiments was determined by gel electrophoresis. Filtered RNA stock solutions were loaded on a 3% agarose gel along with several oligonucleotide standards of known length and the oligonucleotides were visualized with ethidium bromide. On the basis of comparison to the oligonucleotide standards, the yeast RNA used in these experiments appears to contain between 30 to 50 base-pairs.

Full length short-strand RNA was quantified by binding of RiboGreen (Invitrogen), which fluoresces when bound to RNA. This RNA quantification method was used instead of UV-Vis absorbance measurements for detection of ^•^OH-induced RNA degradation for several reasons. Reaction of ^•^OH with RNA leads to alteration of the bases as well as cleavage of the RNA strands. While UV-Vis absorbance is suitable for determining total nucleic acid base concentrations and inferring nucleic acid concentrations, it is not as sensitive as using RiboGreen for measuring short-strand RNA reacted with ^•^OH. This is because cleavage of short-stand RNA will result in a larger fluorescence reduction with RiboGreen compared to the reduction in light absorbance. RiboGreen fluorescence has been found to be relatively independent of RNA fragment size when strands are 500 bases to 9000 bases in length, but declines with decreasing fragment size for smaller fragments: for example, compared to the intensity of fluorescence from 500 base fragments, a 28% drop in fluorescence was noted for 100-base RNA fragments [50]. Hence, by deploying short-strand RNA we capitalize on the sensitivity of the probe with respect to strand-length. The use of RiboGreen also avoids the interference from dissolved transition metal ions in the UV wavelength range, which compromises determinations of RNA concentrations by UV absorbance measurements. Since the concentration of dissolved metals often increases as minerals partially dissolve, this contribution to the UV absorbance rises and is often difficult to correct for.

The experiments in this study were conducted according to a protocol described in previous work [49]. RiboGreen, stored at -20°C, was thawed and diluted 200 × with water (Easy Pure 18.3 MΩ-cm, UV-irradiated, ultrafiltered) in triethanolamine (TE) buffer (Invitrogen). Coal samples were weighed into opaque 50-mL centrifuge vials so that the total surface area of each mineral sample was 0.125 m^2^/L. 1.5 mg/L RNA solutions were added to the vials to initiate the experiments. The vials were placed in an orbital shaker (Thermolyne) operating at 320 rpm at room temperature (22 ± 2°C). Samples were withdrawn from the suspensions with syringes at various times and immediately filtered through 0.45 μm PVDF (Millipore) filters upon collection. 1 mL samples of the filtrates were added to a 4-mL fluorescence cuvette with 1 mL of diluted RiboGreen dye. The cuvettes were kept in the dark and the fluorescence (excitation/emission 490/520 nm) recorded after three minutes.

## Results

**Figure 2:**
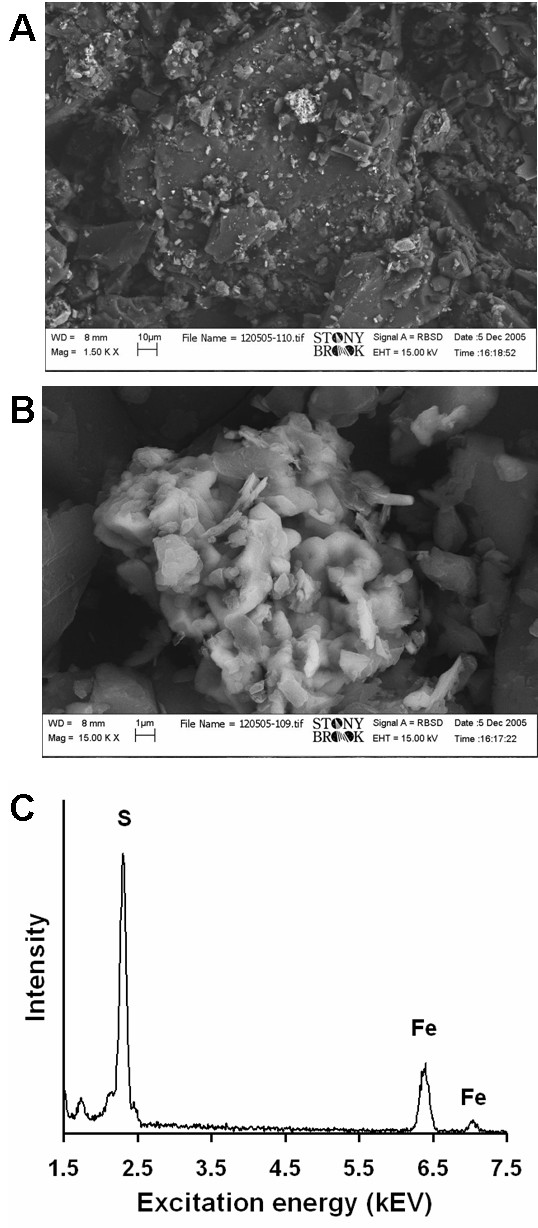
Scanning electron microscopy (SEM) and energy-dispersive X-ray analysis (EDAX) of the coal sample containing the most sulphur (NIST # 2685b). The top image **A **shows grains of carbonaceous material with a cluster of 'bright' crystals. Image **B **is shows the 'bright' crystals magnified and an EDAX spectrum focused on the crystals reveals the presence of iron and sulfur (**C**). An equivalent spectrum was recorded on a pyrite sample (not shown). An energy of 15 kV and a working distance of 8 mm were used for both **A **and **B**, 1500 × and 15000 × magnifications were used for **A **and **B**, respectively.

An SEM image and EDAX analysis of the coal sample that contains the highest concentration of sulfur and iron (NIST #2685b) shows the presence of small platy grains of FeS_2 _(Fig. 2). The composition of the grains, as measured with EDAX, indicates a composition consistent with 1:2 iron to sulfur abundance ratio. An EDAX analysis of pure pyrite (not shown) has the same peak height ratio as in the sample. The platy morphology of the crystals suggests that most of the FeS_2 _may be present as marcasite (a dimorph of pyrite). While pyrite is typically present as cubes or octahedra, marcasite often assumes a tabular morphology [51].

**Figure 3:**
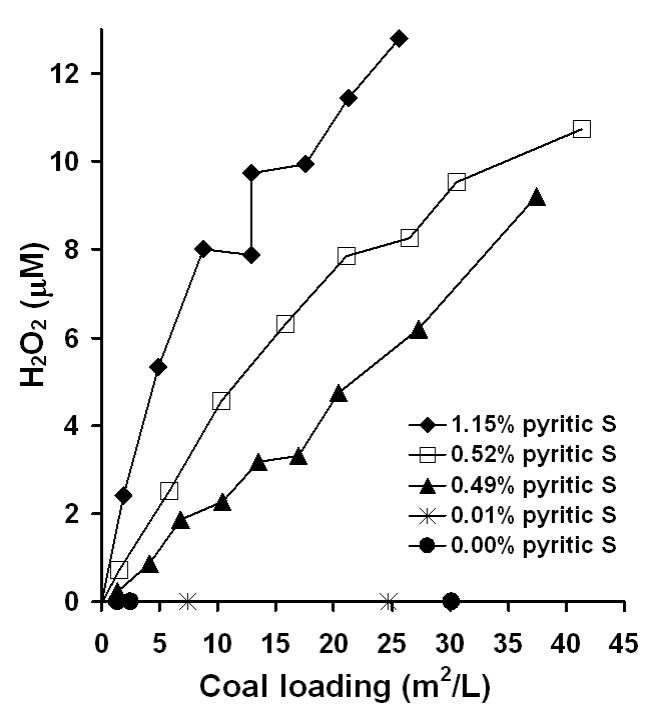
H_2_O_2 _generation from coal samples of varying sulfur content (i.e., FeS_2_) in the presence of 1 mM EDTA. EDTA solutions were mixed with dry coal samples for about 2 min, filtered, leuco crystal violet (LCV) assay agents added, and absorbance at 590 nm recorded. LCV oxidation by H_2_O_2 _results in a colored product, which scales with H_2_O_2 _concentration. A 30 m^2^/L total surface area of each of the coal samples was achieved at mass concentrations ranging from 6 g/L to 26 g/L depending on the physical properties of each coal sample.

Experiments were performed to evaluate the generation of hydrogen peroxide from coal/water suspensions. Coal/water suspensions generate H_2_O_2 _and the amount increases as a function of both coal loading based on surface area and sulfur content (Fig. 3). EDTA was added to the solutions to stabilize H_2_O_2_, which would have otherwise reacted with ferrous iron (eq. 3). With the addition catalase and EDTA (or without EDTA), no H_2_O_2 _was detected. H_2_O_2 _measurements were taken from several coal/water suspension vials that have been mixing from 2 to 30 minutes prior to being filtered followed by addition of the LCV reagents. The H_2_O_2 _concentration is highest after around 2 to 3 minutes, after which the H_2_O_2 _concentration drops about 90% in 15 minutes and H_2_O_2 _is not detected after 30 minutes (data not shown). Results in Figure 3 were taken after 2 minutes of mixing the coal particles with water and EDTA prior to addition of LCV reagents. The results show a correlation between H_2_O_2 _generation and pyrite content of the coal. In addition, H_2_O_2_-generation scales with surface area suggesting that the mechanism of H_2_O_2 _formation is surface dependent. The samples with the lowest sulfur and iron content (i.e., 0.36 to 0.49 % sulfur by mass) show no H_2_O_2 _formation.

**Figure 4:**
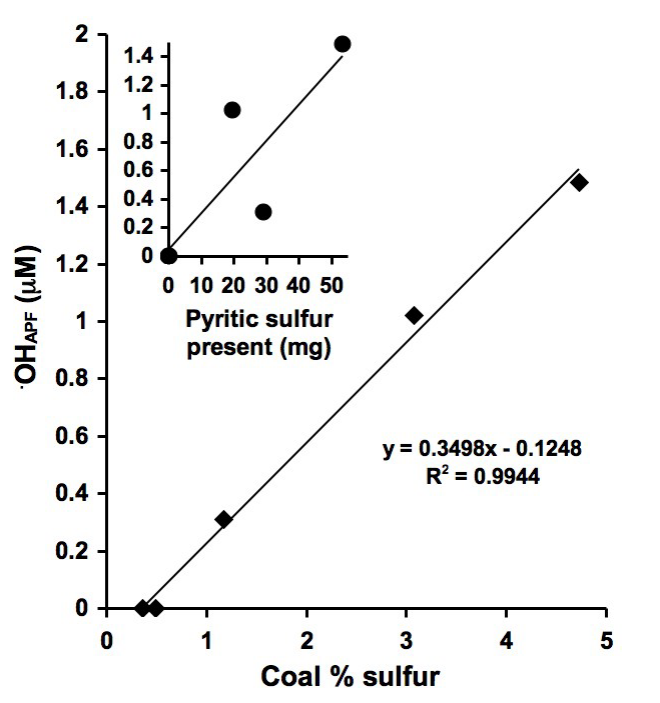
OH generation from coal/water slurries using APF. 40 m^2^/L coal loadings were mixed with an APF solution for 24 hrs followed by filtration and fluorescence measurements. The insert shows ^•^OH formation as a function of total pyritic sulfur present in the coal samples. Fluorescent intensity has been converted to ^•^OH_APF _by a factor determined by reacting APF with known concentrations of H_2_O_2 _and HRP.

**Figure 5:**
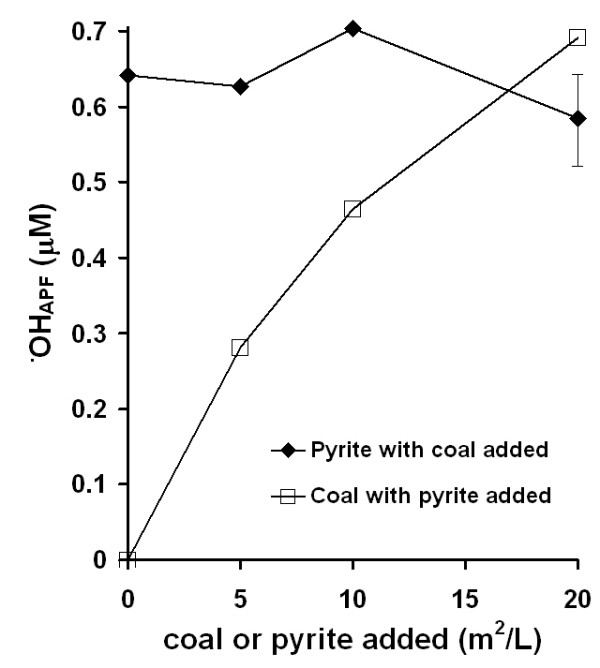
OH generation comparison between the organic carbon and FeS_2 _fractions of coal, measured with APF. In both experiments, APF was exposed to 20 m^2^/L particle loadings with additions of either coal (NIST #1635, coal that does not produce detectable levels of ^•^OH) or pyrite for 24 hrs in the dark followed by fluorescence measurements. In the top curve, pyrite was mixed with incremental additions of coal and in the bottom curve coal was mixed with incremental additions of pyrite. The last two points are repeat experiments. A typical error bar is included on one of the points to show that their standard deviations overlap. Fluorescent intensity has been converted to ^•^OH_APF _by a factor determined by reacting APF with known concentrations of H_2_O_2 _and HRP as in Figure 4.

Separate experiments were performed using APF to quantify generation of ^•^OH in coal/water suspensions. Results show that coal/water suspensions (in absence of EDTA) generate ^•^OH and the quantity of ^•^OH formed scales with coal sulfur content (Fig. 4). To compare the roles of the organic carbon versus FeS_2 _components of the coal in generating ^•^OH, two types of experiments were performed, (a) progressively increasing quantities of low-S coal (#1635), which failed to generate any ^•^OH when added alone to water, were added to a fixed quantity of pyrite (Huanzala, Peru) in water without EDTA and (b) progressively increasing quantities of the same sample of pyrite were added to a fixed quantity of low-S coal under the same conditions (Fig. 5). Incremental additions of the coal sample to a fixed quantity of pyrite do not significantly alter the levels of ^•^OH detected by APF. However, when quantities of pyrite are added incrementally to a fixed amount of low-S coal, the levels of ^•^OH as detected by APF are observed to increase. An estimate of the uncertainty in the APF method as applied to these coal/pyrite slurries is expressed as a 1-sigma error bar for the final point of the series with constant pyrite and variable coal content. The final points of both series have exactly the same composition. Therefore, the measured ^•^OH_APF _concentrations from both samples are within error.

**Figure 6:**
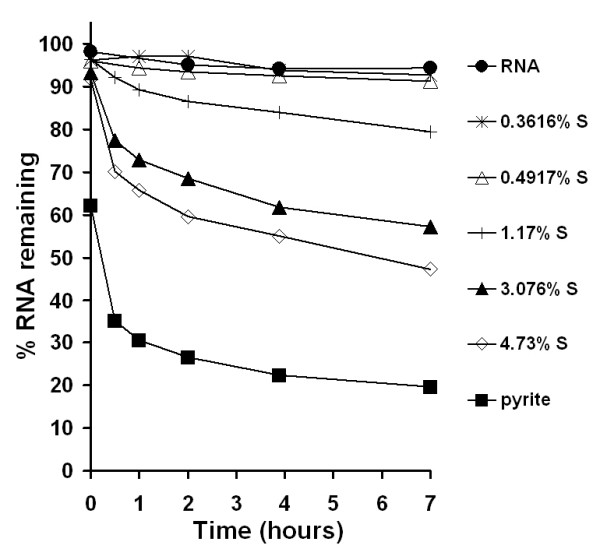
Yeast RNA degradation in the presence of 0.125 m^2^/L coal particle loadings (25 to 107 μg/mL depending on surface area). Coal samples of varying pyritic sulfur content were exposed to 1.5 mg/L RNA, which was quantified using fluorescence of RiboGreen dye. Dissolved iron concentrations were measured at the end of the experiment (i.e., 7 hours) and are listed in Table 1. At the end of the experiment the pH of the RNA solution was 5.64; the pH of coal samples ranged from 5.00 to 5.52; and the pH of the pyrite sample was 4.87. Previous studies with yeast RNA exposed to pyrite have shown that the apparent RNA degradation is not due to a reduction in pH of solution.

Coal samples that contain pyrite degrade RNA (Fig. 6). In these experiments, an equivalent surface area of each coal sample (0.125 m^2^/L) was exposed to a 1.5 mg/L yeast RNA solution and the RNA was periodically quantified for a duration of 7 hours. Results from earlier studies using gel electrophoresis show that pyrite causes RNA strand cleavage [24]. Here, cumulative RNA fragmentation increases as a function of time and pyrite content in the coal. The coal samples that contain 0.00 to 0.01% pyritic sulfur by mass show no RNA degradation.

## Discussion

The formation of ^•^OH and H_2_O_2 _from coal samples that contain pyrite is consistent with our previous finding that pyrite can spontaneously generate ^•^OH [24] and H_2_O_2_[33]. The lack of ^•^OH and H_2_O_2 _from coal samples that do not contain pyrite implies that the mechanism involves pyrite and not the organic fraction of coal. The main experimental observations are listed below.

• The sulphur content of coals can be correlated with ferrous iron release into solution

• Some of the iron content of coal can be correlated to an FeS_2 _phase

• Coals containing < 1% sulphur form no detectible H_2_O_2 _or ^•^OH

• Coals containing < 1% sulphur do not degrade RNA

• Coals containing > 1% sulphur form H_2_O_2 _and ^•^OH and degrade RNA

• The concentration of sulphur in coal samples can be correlated to:

• Concentrations of H_2_O_2 _and ^•^OH formed in solution

• Rate of degradation of RNA

**Figure 7:**
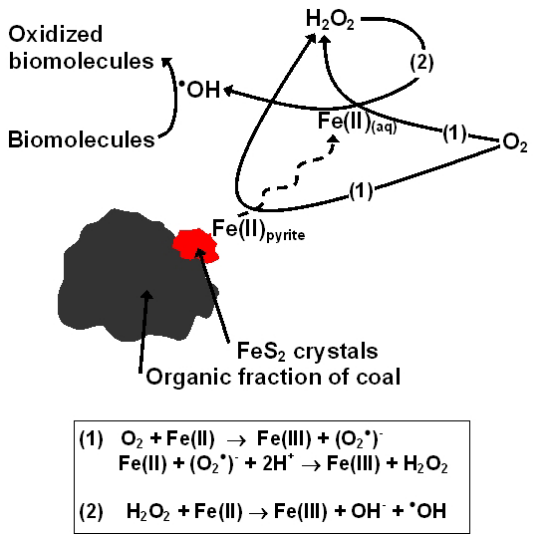
Mechanisms whereby coal that contains pyrite reacts with dissolved oxygen to generate H_2_O_2 _and ^•^OH, with ^•^OH leading to biomolecule degradation. In this diagram, dissolved oxygen reacts with either ferrous iron at the pyrite surface or dissolved Fe(II) to form H_2_O_2 _through the Haber-Weiss reactions (1), which may react with dissolved Fe(II) to form ^•^OH through the Fenton reaction (2).

The mechanisms whereby coal that contains pyrite generates H_2_O_2 _and ^•^OH are schematically presented in a diagram (Fig. 7). In this diagram, dissolved oxygen reacts with either ferrous iron at the pyrite surface or dissolved Fe(II) to form H_2_O_2 _through the Haber-Weiss reaction, which may react subsequently with dissolved Fe(II) to form ^•^OH through the Fenton reaction. It is ^•^OH which reacts with and degrades biomolecules such as nucleic acids, lipids, and proteins. A reaction arrow has been drawn from the ^•^OH to the coal surface because it remains unknown whether the organic fraction of coal reacts with ^•^OH. Whether the mechanism(s) whereby coal that contains pyrite involve reactions at the pyrite surface, reactions in solution, or a combination of surface and solution-mediated reactions remains unknown. Huang et al. have shown a correlation between the prevalence of CWP and release of ferrous iron into in an acidic solution from coal samples [25]. While ferrous iron dissolved into solution may lead to ^•^OH formation, reaction of ferrous iron at the pyrite surface with oxygen may also lead to the formation of ^•^OH. We speculate that the toxicity of coals that contain pyrite is not only related to release of ferrous iron into solution but also reactivity at the pyrite surface. In fact, earlier work by our group shows that the presence of solid pyrite promotes the rate of RNA decomposition, exceeding the RNA decomposition rate in the presence of dissolved ferrous iron alone. Experiments in the presence of pyrite lead to a more rapid degradation of RNA, even though the dissolved iron concentration is comparable in both experiments [24].

The formation of ^•^OH scales strongly with the sulfur content of the coals when the coals are evaluated on an equal surface area basis (Fig. 4). However, as a result of using different particle loadings to ensure equal surface area, the mass of pyrite present in each coal/APF suspension does not increase linearly (see *Pyritic sulfur content per surface area *column in Table 1). The results are consistent with earlier work which showed that the pyrite-induced ^•^OH generation mechanism scales with pyrite surface area [24], which strongly implies a surface-mediated mechanism.

Experiments were performed to evaluate any additional contribution of ^•^OH formation from the organic carbon fraction of coal in addition to the contribution of the FeS_2 _fraction that we have documented here. The results show that generation of ^•^OH is dependent on the FeS_2 _content, but independent of the organic carbon content. This suggests that the organic fraction in the coal tested here is neither a sink nor a source of ^•^OH under the conditions of these experiments. This observation may not extend to the organic fraction or inorganic fractions other than pyrite in other coals. The composition of the organic fraction of coal varies with rank and origin. Hence, a study with a broader sample set is justified. Furthermore, it should be noted that while the organic fraction in the coal tested here is unreactive under the conditions of the batch experiments, this may not be the case when the materials are taken up by cells or otherwise interact with cell-derived products.

Experiments with yeast RNA show degradation only in the presence of coal that contains pyrite and the rates of degradation scale with pyrite content (Fig. 6). The degradation of RNA in the presence of coals that contain pyrite is consistent with earlier studies [24] where pyrite-generated ^•^OH resulted in decreased RiboGreen binding upon exposure to pyrite. There is no evidence that RNA is adsorbing to the pyrite surface, but adsorption of RNA degradation products cannot be ruled out [24].

The results presented in this study show that the pyrite content of coal may be a factor that contributes to the difference in prevalence of lung disease among coal miners in different mining regions. However, it is important to note that additional factors such as size, shape, and the presence of other metals may also contribute to the observed difference in disease prevalence [5-7]. In addition, the scope of the study was limited to a narrow selection of coals that were available from the NIST. Further studies to resolve the role of the interaction between the organic fraction of the coal and pyrite should involve a wider selection of coals and include cell-based assays. By comparing coal samples of varying pyrite content with cellular toxicity and cellular formation of ROS, the role of pyrite and ROS in the mechanisms that lead to pathological conditions could be further understood.

## Conclusion

The ROS generation within the coals studied here is strongly correlated with pyrite content. Addition of natural pyrite to coal devoid of pyrite increases the ROS formation in a predictable way. Hence the organic fraction of the coals studied here does not appear to scavenge pyrite-induced ROS. It is important to note though that the scope of the study was limited to a small set of well-characterized coals available from NIST. Hence, a subsequent study with a broader selection of coals and experiments in which lung cells are exposed to coal with different pyrite content are warranted.

On the basis of the results presented in this study, we hypothesize that the pyrite content of coal is a significant factor in determining the prevalence of lung disease among coal miners. While consistent with the observed regional differences in CWP prevalence and regional differences in sulfur (pyrite) content in the US [25], it is important to note that additional factors such as size, shape, and the presence of other metals (i.e., mercury, nickel, cobalt, and arsenic) in the coal may also contribute to the observed difference in disease prevalence. Some of these additional factors may be directly related to the pyrite content. For example, arsenic is often associated with pyrite in coals [52].
